# Institutional Notes and News

**Published:** 1910-06-18

**Authors:** 


### Institutional Notes and News.

**Edinburgh Maternity Hospital.**

In the last five years the patients at the Edinburgh Royal Maternity and Simpson Memorial Hospital have more than doubled. Last year no fewer than 1,914 persons were treated. This number accounts for the excess of expenditure over income which was noticed in the hospital's annual report. The income amounted to £1,825, and the expenses to £2,522. This state of affairs has led the governors to consider what plan should be adopted to deal with the growing work of the institution, and finally to give to the city a hospital for obstetric cases equipped fully with up-to-date appliances. Sir Robert Cranston presided at the recent annual meeting and remarked that the maternity hospital was doing work which no other institution was doing equally well, and consequently that it put forward an appeal for money to carry out the necessary improvements, which a tendency to cramp the patients had created, with some confidence. The nurses, too, Sir Robert pointed out, wanted much better accommodation. "I think," he went on, "all such institutions should, as in Germany, have a grant—so much from the State and so much from taxes. The taxes are quite heavy enough." He also thought more might be done by the University. Mr. M'Ewen also emphasised these points, adding that the weight of taxation had diminished the number of subscribers, and that the problem of extending the hospital had been in consideration for some little time.

**Oxford Eye Hospital.**

The Oxford Eye Hospital has approved the plans for building a new room for pathological work and *x*-ray purposes, without which the hospital is felt to be incomplete. Before the building can be proceeded with, however, the approval of the Radcliffe Infirmary must be gained, as the Radcliffe is the lessor, without the consent of which no addition to the present buildings can be made. Surgeon-General Bradshaw, the honorary secretary, said that the work of publishing the hospital's report had been going forward steadily, and that 1,600 copies had been printed and distributed, first to subscribers, and secondly to the newspapers. General Bradshaw mentioned also the retirement of several members of the committee, who for one reason or another had been prevented from attending its meetings. This is a tiresome year for the Oxford Eye Infirmary, in that the repainting of the exterior is due, a necessary but uncomfortable piece of work which makes generally the three years' interval between each repainting seem unpleasantly short to all concerned.

**Cottage Hospital, Easingfold.**

The transfer of St. Monica's Cottage Hospital, Easingfold, Yorkshire, from private to public hands is now an accomplished fact. That efforts were being made for this purpose was mentioned in the columns of our "Hospital World" last week. Mrs. Love, who has supported the hospital herself hitherto, has given the institution to the neighbourhood, which has undertaken to maintain it. The next thing to do therefore was to elect a committee and draw up the rules. We take from them one which refers to the patients' obligations to pay : "Patients shall contribute to the funds of the hospital not less than 2s. 6d. each week. Subscribers shall be requested to state on the recommendation form what sum they consider the patient able to pay; and the patient or relation shall be required to undertake that such sum shall be paid weekly to the matron. The payment may be remitted by the Executive Committee in exceptional cases. Private patients, subject to the approval of the Executive Committee, can be received at a fixed rate of not less than £1 1s. per week. Domestic servants and resident farm servants can only be received as private patients at the following rates, namely :—If from houses the rental of which is over £50, at £1 1s. per week; under £50, at 10s. 6d. per week; and under £20, at 5s. per week, exclusive of medical attendance. One bed shall be reserved free for non-paying patients recommended by the Lady President." The district has proved so ready to subscribe the initial sum needed that the hospital's future support should never be wanting.

**Glasgow Dental Hospital.**

A quarter of a century of work has been completed by the Incorporated Glasgow Dental Hospital, and these twenty-five years have revealed two important things. The first is that dentistry has improved considerably in the interval since, in the words of the report, "the quality of the services rendered by the staff has been reflected in the increasing number of conservative operations as compared with extractions," showing that "toothache" is being treated scientifically at last. The second is that the public has been educated enough to grasp the fact that, in the words of Sir James Fleming, "neglect of the teeth and diseases of the teeth not only cause pain and suffering, but really affect the general health." The recognition of this has been long in coming, and now it has come the hospitals are getting rather alarmed at the number of dental and ophthalmic cases which the medical inspection of school-children is sending to their doors. Still, we suppose it is an exaggeration on the right side to say that the teeth of the poor are never sound, and it is certainly true that this unsoundness is due to early neglect. Then comes the question of the remedy, and what that remedy costs. Sir James Fleming naturally alluded to it, pointing out that 14,362 cases had been treated (which was 1,000 more than in the previous year), and that therefore the hospital was doing "a service to the community that the latter ought really to pay for." Some fifty students attend the hospital, and but for their fees the hospital—with the additional land it has purchased recently for the extension that is looming ahead—could not be carried on. These facts should give tremendous driving force to an appeal, which seems to be needed judging from the fact that "the (annual) subscriptions by the public are over £30 less than they were during the first full year of the hospital's history."

**Albert Infirmary, Winsford.**

The Albert Branch Infirmary, Winsford, a branch of the Victoria Infirmary, Northwich, Cheshire, now possesses a new operation unit, which was opened last week by the Countess Grosvenor. The original theatre had done duty for twelve years, since the hospital's foundation, in fact; and as ninety-two operations were performed last year, the need for a new one was obvious. The old theatre was cramped and out of date, but it was feared that there would be some difficulty in raising money enough to pay for a new one. The cost has proved to be £826, including the furnishing, and this sum has been raised in twelve months, which is a credit to everyone's energy, and particularly, perhaps, to that of the hospital Secretary, Mr. J. H. Cooke. The unit consists of the anæsthetist's room, the theatre proper, and a sterilising room. The unit stands between the women's ward and the kitchens, and under the same roof, though quite separate from the theatre, are to be found the bathroom and lavatories for the women's ward, which, of course, are entered from it. The details of equipment were entrusted to a sub-committee of three medical men, so that there is every reason to believe that these are satisfactory. Owing to an enthusiastic spurt at the last moment not only has the whole cost been paid, but there is a balance of £34 in hand.

**Children's Hospital, Shirley.**

Early this month a subscribers' meeting of the Children's Hospital and Dispensary for Women, Shirley, Hampshire, was held to discuss the hospital's future. In the words of the Chairman of the Managing Committee, Captain J. Henderson, R.N., a crisis had come in the affairs of the Shirley Hospital. The house was for sale, and, with the increase in the working-class population, there had been an increased number of women and children requiring medical and surgical aid. The Committee was aware of the inadequacy of the premises, and had considered various courses to obtain a solution of the difficulty. One was to remain where they were, and purchase the place, doing the best they could with it. They were in a position to do that, as, fortunately, they had sufficient money to pay for the house. Presuming they bought the house, they would want £450 to make the alterations which were absolutely necessary. Then the Committee had been looking round to see whether there was another house suitable, and the only place they had found likely to suit was a place called Calvados, a fine house in Anglesea Road. They had had an offer from the owners to let them have it at a reasonable sum. Difficulties had cropped up as to taking the place. The question was whether they could raise enough to buy Calvados, alter it, and pay for the extra nursing. If they could the place was admirably fitted for a hospital. After some discussion the Chairman's motion that the Committee be empowered to treat for the purchase of Calvados, and, failing such purchase, to ensure the continuity of the work carried on in the present hospital was carried.

**Bristol Royal Infirmary.**

The extension scheme for the Bristol Royal Infirmary was decided on last Tuesday. It will cost £70,000, half of which sum is now in hand, and will take the form of a new wing for 180 surgical beds. There will be a reduction of a hundred beds in the old building, giving, however, a total of 350, or a net gain of eighty over the existing number of 270. Sir George White, the hospital's president, said that this was better than any plan for cobbling the old building. Mr. Samuel White, Sir George's brother, said that if £30,000 of the £35,000 required were subscribed within twelve months he would willingly himself give the remaining £5,000. Mr. White suggested also that the new wing should be made a memorial to King Edward, which proposal was endorsed by Sir George, who said that there could be no better way of perpetuating King Edward's memory in Bristol. It should be added that the total cost of £70,000 is made up as follows:—For the site of the new wing, £15,000; for the actual building, £50,000; for certain necessary expenses in the old building, £5,000. If the generosity of Bristol is at all equal to that of Mr. Samuel White there should be no delay in raising this sum, and Bristol is a city great enough to afford to be generous in a good cause.

